# Effects of low-serum adaptation on growth and protein expression in stably lactoferrin-overexpressing mammary alveolar cells

**DOI:** 10.3389/fbioe.2026.1731548

**Published:** 2026-01-15

**Authors:** Zhenxing Qiang, Xiaoqian Cai, Zhenzhen Zhang, Hui Wang, Qiuying Wang, Yajing Ji, Guangpeng Li, Guanghua Su, Lei Yang, Weicang Qiao, Junying Zhao, Ming Chen, Chunling Bai, Lijun Chen

**Affiliations:** 1 School of Bioengineering, Dalian Polytechnic University, Dalian, China; 2 College of Life Sciences, Inner Mongolia University, Hohhot, China; 3 National Engineering Research Center of Dairy Health for Maternal and Child, Beijing Sanyuan Foods Co. Ltd., Beijing, China; 4 Beijing Engineering Research Center of Dairy, Beijing Technical Innovation Center of Human Milk Research, Beijing Sanyuan Foods Co., Ltd., Beijing, China

**Keywords:** cell growth, lactoferrin, low-serum adaptation, MAC-T cells, protein expression

## Abstract

**Objective:**

Long-term culture with high serum concentrations increases culture costs, accelerates cell senescence, and limits industrial-scale applications. Serum reduction adaptation is an effective solution for addressing these issues. In this study, we aimed to investigate the effects of two low-serum adaptation strategies on the growth and lactoferrin expression in stably LTF-overexpressing bovine mammary alveolar cells transfected with large T antigen (MAC-T).

**Methods:**

Two adaptation protocols of serum concentration reduction and dual-medium gradient adaptation were implemented to progressively reduce serum dependence in MAC-T cells.

**Results:**

Under both adaptation modes, cells maintained stable proliferation at 1% fetal bovine serum (FBS). At the 0.5% FBS (serum reduction) and 10% basal control medium, equivalent to 1% FBS in dual-medium adaptation stages, adding 10% knockout™ serum replacement and 1% insulin-transferrin-selenium 5 increased the specific growth rate by 30%, enabling robust cell growth under low-serum conditions.

**Conclusion:**

Across all the low-serum conditions, cells retained typical epithelial morphology (cytokeratin 18-positive) while exhibiting consistent *LTF* mRNA transcription levels, protein expression, and secretory output. These findings validate the scalable low-serum culturing of MAC-T cells and *in vitro* LTF synthesis.

## Highlights

- Low-serum adaptation reduces costs while sustaining MAC-T cell growth.
- Serum reduction and dual-medium strategies support stable proliferation.
- Growth rate rose 30% with serum replacement + insulin-transferrin-selenium.
- Cells preserved morphology and consistent lactoferrin expression *in vitro.*

## 1 Introduction

Lactoferrin (LTF) is a natural cationic iron-binding glycoprotein, which is expressed and secreted by the mucosal epithelial cells of various mammals (Faraji et al., 2017). In addition to regulating iron ion absorption and immune responses, LTF exerts diverse biological activities, including antibacterial, antiviral, antioxidant, anticancer, and anti-inflammatory properties, which underscore its broad application prospects. (Rastogi et al., 2016; Wu et al., 2024; Eker et al., 2024; Rascón-Cruz et al., 2024; Chandran et al., 2025). In terms of researches on host expression of lactoferrin, prokaryotic systems, such as *Escherichia coli*, tend to form inclusion bodies, which results in complex renaturation processes and difficulty in ensuring activity (García-Montoya et al., 2013). Furthermore, a major limitation of eukaryotic yeast systems is that their protein glycosylation modification differs from the native structure (Lv et al., 2024). Both systems are unable to meet the requirements for high-quality preparation. However, mammary alveolar cells relying on a suitable eukaryotic expression environment enable to express lactoferrin with a closer structure to the natural form and precised post-translational modifications. This phenomenon substantially enhances the functional adaptability of the protein. Therefore, mammary alveolar cells are a key research focus in industrial production directions, such as cell reactor applications.

In biopharmaceutical industry and cell culture, the dependence of mammalian cells on serum limits its process stability, cost control, and scaled-up production (Sattari et al., 2024). Generally, serum is crucial for cell adhesion, proliferation, and maintenance of vital activities because it contains multiple essential components and abundant nutrients (Fang et al., 2017; van der Valk et al., 2018; Subbiahanadar Chelladurai et al., 2021). Traditional high-serum culture systems effectively support cell proliferation; however, they suffered from inherent limitations, including undefined chemical composition, significant batch-to-batch variability, potential viral contamination risks, and considerable cost burden. This accounts for over 50% of the total cost of the culture medium, which conversely prompted the development of low-serum acclimation technologies. (Chou et al., 2015; Hawkes, 2015; Baker, 2016; Kolkmann et al., 2020). Low-serum culture systems gradually reduce serum concentration in the culture medium to induce cellular adaptation to a nutrient-limited microenvironment, thereby enhancing process standardization, facilitating the transition toward serum-free culture, and providing robust technical support for the production of vaccines, antibodies, and recombinant proteins (Ali et al., 2025). With the escalating safety and quality requirements for biological products, acclimation which characterized by the gradual reduction of serum concentration has been increasingly promoted. This process enables cellular adaptation to low-serum or serum-free environments, thereby enhancing production efficiency and mitigating potential risks. (Stout et al., 2022).

Serum reduction strategies for cell adaptation usually applied two primary methods, the gradual stepwise reduction of serum concentration and the stepwise adaptation using mixed media formulations. Previous studies have validated the distinct characteristics of these two approaches: the gradual stepwise reduction method achieves adaptation by sequentially decreasing serum concentration. It is cost-effective as it eliminates the need for complex medium preparation, but often requires multiple passages (4–8 weeks) and may cause significant fluctuations in cell viability, resulting in reduced yield of target proteins (Arzu et al., 2020). In contrast, the mixed media gradient method provides a gentler environmental transition, minimizes cellular stress during adaptation, and maintains normal cellular morphology and functional stability. However, this method necessitates precise optimization of medium mixing ratios, increasing operational complexity and medium costs (Wu et al., 2021). These approaches are supplemented with serum replacements, such as knockout™ serum replacement (KSR) and insulin-transferrin-selenium (ITS) to enhance cellular adaptability (Zhang et al., 2006; Garcia-Gonzalo and Izpisúa Belmonte, 2008). However, most existing studies focus on a single adaptation strategy rather than conducting direct comparisons under the same experimental system, making it difficult to identify the optimal approach that balances cost and efficacy. Therefore, in this study, we compared the effectiveness of these two serum reduction adaptation methods using a MAC-T cell model and analyzed the impacts of the serum reduction methods on cellular growth and LTF expression stability. This study provided theoretical and experimental foundations for establishing an efficient and stable low-serum culture system.

## 2 Materials and methods

### 2.1 Materials

Bovine mammary epithelial MAC-T cells were purchased from Shanghai Jinyuan Biotechnology Co., Ltd. Dulbecco’s Modified Eagle’s Medium, serum-free medium (SFM), FBS, Knockout™ serum replacement (KSR), Pen-Strep solution, trypan blue, trypsin-EDTA, and ITS were obtained from Gibco, Thermo Fisher Scientific. The phosphate-buffered saline (PBS), bovine serum albumin (BSA), and epidermal growth factor (EGF) were purchased from Sigma-Aldrich, Merck. RNA-easy isolation reagent, HiScript II Q RT SuperMix for qPCR, ChamQ Universal SYBR qPCR Master Mix, and 2×Taq Master Mix were obtained from Vazyme Biotech. LTF detection kit based on enzyme-linked immunosorbent assay (Cloud-Clone Corp, Wuhan, China), ExpressPlus™ PAGE Gel (GenScript Biotech), horseradish peroxidase-conjugated Affinipure goat anti-rabbit IgG (H + L), Multi-rAb™ CoraLite® Plus 594-Goat anti-rabbit recombinant secondary antibody (H + L), lactoferrin polyclonal antibody, *GAPDH* polyclonal antibody (Proteintech), and cytokeratin 18 (CK18) antibody (Bioss) were purchased from the corresponding reagent companies.

### 2.2 Adaptation to low-serum conditions and growth of LTF-overexpressing MAC-T cells

#### 2.2.1 Culture procedures for serum step-down and dual-medium gradient adaptations

**FIGURE 1:**
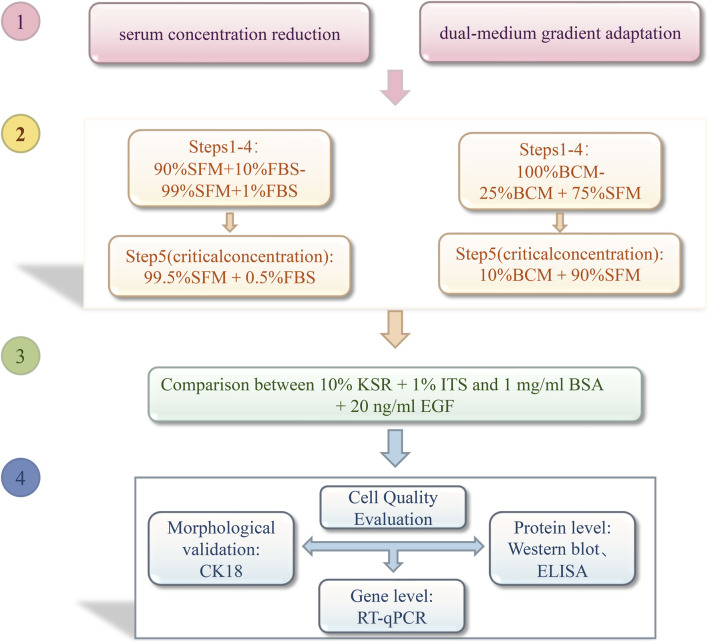
Flowchart of serum adaptation strategies. Serum concentration reduction culture: Based on the SFM, the serum proportion was directly reduced from 10% to 0.1% in the sequence 10%, 5%, 2%, 1%, 0.5%, and 0.1%. Dual-medium gradient culture: This culture was prepared by adjusting the serum ratio of the BCM basic medium prepared with Dulbecco’s Modified Eagle’s Medium (9:1 → 7:3 → 5:5 → 3:7 → 1:9). The serum step-down and dual-medium gradient adaptation methods followed a regimen involving three consecutive passages at a split ratio of 1:3 per adaptation step. BCM: Dulbecco’s Modified Eagle’s Medium +10% FBS + Puromycin (Puro).SFM: serum-free medium. KSR: Knockout™ serum replacement. ITS: insulin–transferrin–selenium. BSA: bovine serum albumin. EGF: epidermal growth factor.

According to the adaptation protocol (Figure 1), the serum step-down and dual-medium gradient adaptation methods followed a regimen involving three consecutive passages at a split ratio of 1:3 per adaptation step. Based on the serum medium formulations for different adaptation steps outlined in Table 1, cells were subjected to a gradual serum reduction procedure. This was achieved by culturing the cells in media prepared with varying serum proportions, including the basal control medium (BCM: Dulbecco’s Modified Eagle’s Medium +10% FBS + Puromycin (Puro)) and SFM. During the adaptation process, cell viability (>90%), population doubling time, and specific growth rate were monitored to ensure that the cells adapted to the progressively reduced serum conditions and exhibited stable growth (Jang et al., 2022).

**TABLE 1:** Formulation of serum medium for different low-serum steps.

Stepwise serum reduction acclimation procedure	Dual-medium gradient adaptation	Serum concentration reduction
Step 1	100% BCM	90% SFM +10% FBS
Step 2	75% BCM +25% SFM	95% SFM +5% FBS
Step 3	50% BCM +50% SFM	98% SFM +2% FBS
Step 4	25% BCM +75% SFM	99% SFM +1% FBS
Step 5	10%BCM +90%SFM	99.5% SFM +0.5% FBS
Step 6	1%BCM +99%SFM	99.9% SFM +0.1% FBS
Step 7	100% SFM	100% SFM

Dual-medium gradient adaptation: This method involved the combination of the basal control medium (BCM) prepared in Dulbecco’s Modified Eagle’s Medium and a commercial serum-free medium (SFM). Cells were adapted using media formulated by mixing these two base media in varying proportions. Serum concentration reduction: This method involved a single SFM, base. Adaptation was achieved through a stepwise reduction of serum supplementation, specifically formulated at concentrations of 10%, 5%, 2%, 1%, 0.5%, and 0.1%.

Cell samples were collected by every 24 h. Using the trypan blue staining method, the cell suspension was mixed with the trypan blue solution in a 1:1 volume ratio and added to the standard cell counting plate. Data collection and analysis were performed using the IC100 automatic cell counting system. The viable cell density and cell viability (Cv) were calculated as follows:

$$\text{Cv}=X_{v}\;/\;\left(\;X_{v}+X_{d}\;\right)\times 100\%$$

where Xv represents the density of living cells (mL^−1^) and Xd is the density of dead cells (mL^−1^).

The cell doubling time (DT) was determined via regression based on the exponential growth equation:

$$\text{DT}=T\ln2/\ln\left(X_{e}/X_{b}\right)$$

where T represents the incubation time, Xb is the number of cells at the beginning of incubation, and Xe indicates the number of cells at the end of incubation.

$$\text{Specific growth rate }\left(\mu \right)=\text{In }\left(X_{v2}/X_{v1}\right)/\Delta t$$

where μ represents the specific growth rate (d^−1^) and X_v2_ and X_v1_ correspond to the cell density (mL^−1^) at times t2 and t1, respectively. μ > 0 represents the specific growth rate and μ < 0 indicates the specific death rate.

$$\Delta t=t_{2}–t_{1}\left(d\right).$$

When the cell survival rates were <90% during the low-serum process, the cells were returned to the previous low-serum step for further processing. The following steps were initiated when the cells stabilized.

### 2.3 Screening procedure for serum replacements and combinations

#### 2.3.1 KSR concentration screening

At Step 5 of serum step-down (0.5% FBS +99.5% SFM) and dual-medium gradient (10% BCM +90% SFM) adaptation, KSR was supplemented at concentrations of 5%, 10%, and 15%. The specific growth rate, DT, and cell viability were monitored at each KSR concentration to identify the optimal KSR supplementation level for promoting sustained cellular growth under low-serum conditions (Haraguchi et al., 2025).

#### 2.3.2 Combination concentration screening

During the adaptation process, serum step-down (0.5% FBS +99.5% SFM) and dual-medium gradient (10% BCM +90% SFM) adaptation were used. Step 5 was the control group. The following combinations were supplemented: 1 mg/mL BSA +20 ng/mL EGF and 10% KSR +1% ITS. The growth rate was monitored to identify the most stable combination for promoting cellular growth under low-serum conditions (Gomes et al., 2020; Skrivergaard et al., 2023; Ahmed et al., 2023; Zhang et al., 2006).

### 2.4 Cell quality assessment

#### 2.4.1 Monitoring of specific growth rate, DT, and cell viability

Cell quality was assessed during low-serum adaptation for the following groups:

Serum concentration reduction: Step 1: 10% FBS +90% SFM; Step 2: 5% FBS +95% SFM; Step 5 + KSR/ITS: 0.5% FBS +99.5% SFM additional supplementation of (10% KSR +1% ITS).

Dual-medium gradient adaptation: Step 1: 100% BCM; Step 2: 50% BCM +50% SFM; Step 5 + KSR/ITS: 10% BCM +90% SFM additional supplementation of (10% KSR +1% ITS).

The evaluated parameters were specific growth rate, DT, and cell viability as described in 2.2.1 (Jang et al., 2022).

#### 2.4.2 Immunofluorescence

Cells were cultivated at 5 ×10^5^ cells/well in the six-well plate until attaining a 70%–80% confluence. After washed with pre-cooled PBS for 5 min, cells were fixed with 4% paraformaldehyde for 20 min, permeabilized with 0.1% Triton-X-100 for 1 h, and blocked with 5% BSA at room temperature for 2 h. Then primary antibody CK18 (Bioss, bs2043R) diluted at 1:100 was added, followed by overnight incubation at 4 °C and incubation with a fluorescent secondary antibody (Proteintech, RGAR004) at 1:500 dilution in the dark for 2 h. The cells were washed thrice with PBS and stained with DAPI (Beyotime, C1106) (Hu et al., 2016). The test groups and stages were consistent with those in 2.4.1.

#### 2.4.3 Reverse transcription quantitative polymerase chain reaction (RT-qPCR)

Total RNA was extracted using the HiPure Total RNA extraction kit (Magen, R4111-02) according to the manufacturer’s instructions and then reverse-transcribed into cDNA. The primers used are listed in Table 2. The reaction system was set as follows: 10 μL of 2 × Taq Pro Universal SYBR qPCR Master Mix, 0.4 μL of upstream and downstream primers each, 1 μL of cDNA, and Rnase-Free ddH_2_O to make up to 20 μL. Amplification conditions were as follows: 30 s at 95 °C for pre-denaturation; 10 s at 95 °C for denaturation, 30 s at 60 °C for annealing and extension, a total of 40 cycles. At 95 °C for 15 s, at 60 °C for 60 s, and at 95 °C again for 15 s. The *GAPDH* gene was used as the internal reference.

**TABLE 2:** List of quantitative reverse transcription PCR-specific primers.

Gene	Forward primer (5'→3′)	Reverse primer (5'→3′)
*GAPDH*	GAT​GGT​GAA​GGT​CGG​AGT​GAA​C	GTC​ATT​GAT​GGC​GAC​GAT​GT
*LTF*	GTG​GTG​TCT​CGG​AGC​GAT​AG	TAA​CGA​CCG​CGT​GAG​TCA​AA
*CCND1*	GCC​GAG​GAG​AAC​AAG​CAG​ATC​ATC	CAT​GGA​GGG​CGG​GTT​GGA​AAT​G
*BAX*	TGCTTCAGGGTTTCATCC	CTTCAGACACTCGCTCAG

#### 2.4.4 Western blotting

Total protein from MAC-T cells was treated with protein lysis buffer (990 µL of radioimmunoprecipitation assay buffer with 10 µL of phenylmethylsulfonyl fluoride) for 5 min, and centrifuged at 12,000 × *g*, 4 °C for 15 min. Then, 30 µg of the protein sample was boiled for 10 min at 100 °C. This was followed by SDS-PAGE at a constant current of 140 V for 30 min. The target protein band was cut according to the marker size and transferred to an Nitrocellulose (NC) membrane. After being blocked with BSA, the NC membrane was incubated with the primary antibody (Proteintech, 10933-1-AP) at a dilution of 1:1000 at room temperature for 2 h, followed by incubation with the secondary antibody (Proteintech, SA00001-2) at a dilution of 1:10000 for 2 h and three washes with TBST for 10 min each. The super-sensitive luminescence solution was then added to the NC membrane, which was exposed and developed for imaging.

#### 2.4.5 Detection of LTF production

The LTF content was determined by using ELISA kit (Cloud-Clone Corp, SEA780Bo) according to the manufacturer’s instructions.

### 2.5 Data processing

Statistical analysis was performed using GraphPad Prism version 10 (GraphPad software). The experiment was repeated three times. The statistical differences between two groups were compared by the Student’s t-test, while multiple groups comparison was applied by using one-way analysis of variance (ANOVA), followed by Tukey’s *post hoc* test. Data are presented as mean ± SD. For the RT-qPCR analysis. The *GAPDH* gene was used as the internal reference gene for data normalization. The relative expression value of the gene in the sample was determined using the 2^−△△Ct^ algorithm (Pinheiro et al., 2024). To process the Western blotting data, the protein of *GAPDH* was used as the internal reference, and protein gray-scale analysis was performed using ImageJ software. *P < 0.05, **P < 0.01, and ***P < 0.001 were considered statistically significant.

## 3 Results

### 3.1 Establishment of low-serum adaptation strategy and the determination of critical concentrations

#### 3.1.1 Serum concentration reduction

**FIGURE 2:**
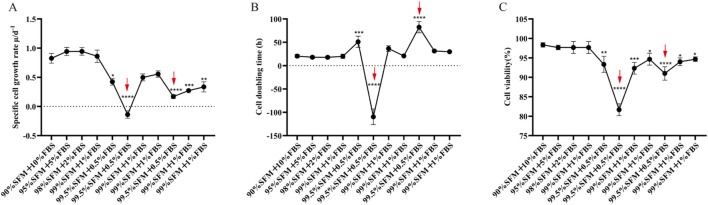
Dynamic data monitoring during serum concentration reduction adaptation of mammary alveolar cells transfected with large T antigen (MAC-T cells). **(A)** Specific cell growth rate; **(B)** Cell doubling time; **(C)** Cell viability. SFM: serum-free medium. *P < 0.05, **P < 0.01, and ***P < 0.001.

During serum concentration reduction (Figure 2), the cells at Step 5 (0.5% FBS) had a significantly lower specific growth rate than those at Step 1 (10% FBS) (P < 0.05), indicating growth deceleration. Meanwhile, the cell viability remained over 90%. To stabilize growth under 0.5% FBS, the cells were passaged three additional times under Step 5 conditions. This resulted in a specific growth rate less than 0% (Figure 2A, red arrow), increased DT (Figure 2B, red arrow), and reduced cell viability (<90%; Figure 2C, red arrow).

These data confirm growth arrest with a trend toward cell death. Therefore, cells were reverted to Step 4 (1% FBS). The cells resumed proliferation under the Step 4 conditions, with the growth rate, DT, and cell viability returning to growth-permissive ranges. After six consecutive stable passages, Step 5 (0.5% FBS) adaptation was repeated and caused significant reductions in specific growth rate, DT, and cell viability (*P* < 0.05) (Figure 2C, red arrows). These imply that Step 5 (0.5% FBS) is the bottleneck concentration for maintaining stable cellular growth in serum concentration reduction.

#### 3.1.2 Dual-medium gradient adaptation

**FIGURE 3:**
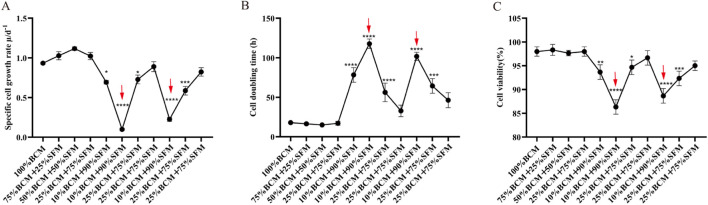
Dynamic data monitoring during dual-medium gradient adaptation of MAC-T cells. **(A)** Specific cell growth rate; **(B)** Cell doubling time; **(C)** Cell viability. BCM: Dulbecco’s Modified Eagle’s Medium +10% FBS + Puromycin (Puro). SFM: serum-free medium. *P < 0.05, **P < 0.01, and ***P < 0.001.

During dual-medium gradient adaptation (Figure 3), the cells at Step 5 (10% BCM, equivalent to 1% FBS) had a significantly lower specific growth rate than those in Step 1 (100% BCM, equivalent to 10% FBS) (*P* < 0.05), indicating slowed proliferation. However, the cell viability remained >90%. To stabilize growth under 1% FBS-equivalent conditions, cells underwent three additional passages at Step 5. These caused the specific growth rate to approach zero (Figure 3A, red arrow), an increased DT (Figure 3B, red arrow), and a reduced cell viability (<90%; Figure 3C, red arrow), confirming growth arrest. Therefore, the cells were reverted to Step 4 (25% BCM, equivalent to 2.5% FBS). Under Step 4 conditions, cells regained normal growth dynamics, with the specific growth rate, DT, and cell viability returning to baseline ranges. Following six consecutive stable passages, re-attempted Step 5 adaptation caused significant decreases in the specific growth rate and cell viability (*P* < 0.001) and a significant increase in DT (*P* < 0.001). This implies that Step 5 (10% BCM, 1% FBS-equivalent) is the critical threshold for maintaining stable cellular growth in dual-medium gradient adaptation.

### 3.2 Optimization of serum replacements and combination screening

#### 3.2.1 KSR concentration screening

**FIGURE 4:**
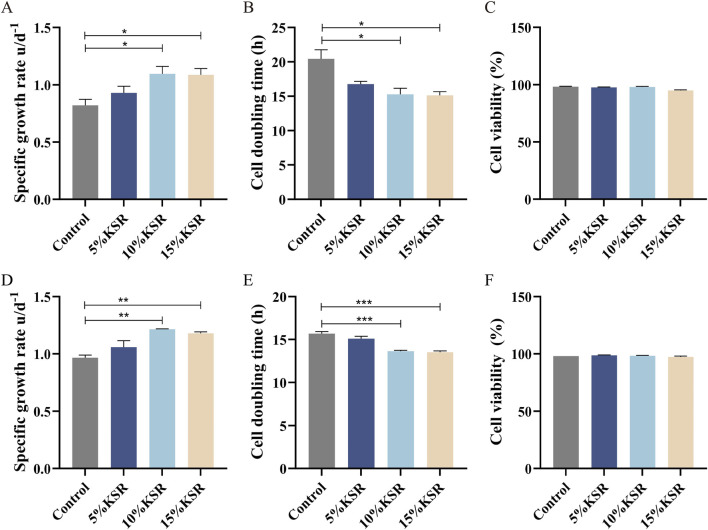
Dynamic data monitoring of the effects of knockout™ serum replacement (KSR) supplementation on cell growth in MAC-T cells. Serum concentration decreasing acclimation at different KSR concentrations: **(A)** Specific growth rate; **(B)** Cell doubling time; **(C)** Cell viability. Two-medium gradient acclimation at different KSR concentrations: **(D)** Specific growth rate; **(E)** Cell doubling time; **(F)** Cell viability. KSR: Knockout™ serum replacement. *P < 0.05, **P < 0.01, and ***P < 0.001.

To promote stable cell growth under low-serum conditions, Step 5 serum concentration reduction (0.5% FBS +99.5% SFM) and dual-medium gradient adaptation (10% BCM +90% SFM) were used as the control groups. Concentrations of KSR (5%, 10% and 15%) were separately added. The specific growth rate, DT, and cell viability among the groups were compared, and the optimal KSR concentration for promoting stable cell growth under low-serum conditions was screened. Under the serum concentration reduction (0.5% FBS) and dual-medium gradient adaptation (10% BCM) Step 5 conditions, the cell viability of all groups (controls and 5%, 10%, and 15% KSR) was over 90% (Figures 4C,F). The specific growth rates of the 10% and 15% groups were significantly higher than those of the control group (*P* < 0.05) (Figures 4A,D). However, their doubling times were significantly lower than those of the control group (*P* < 0.05) (Figures 4B,E). The specific growth rate and DT showed no significant differences between the 5% KSR and control groups. Although 10% and 15% KSR significantly improved the specific growth rate and DT of cells, 10% KSR was more cost-effective. Therefore, 10% KSR was used for subsequent experiments to maintain the growth state of the cells under low-serum conditions.

#### 3.2.2 Comparing combinations of well-defined components

To promote stable cell growth under low-serum conditions, two low-serum adaptation end-point conditions were selected as controls, namely, the Step 5 media in the serum concentration reduction adaptation (0.5% FBS) and dual-medium gradient adaptation (10% BCM, equivalent to 1% FBS) conditions. In the experimental groups, 10% KSR +1% ITS or 1 mg/mL BSA +20 ng/mL EGF were added to the corresponding Step 5 medium of the above control groups, respectively.

**FIGURE 5:**
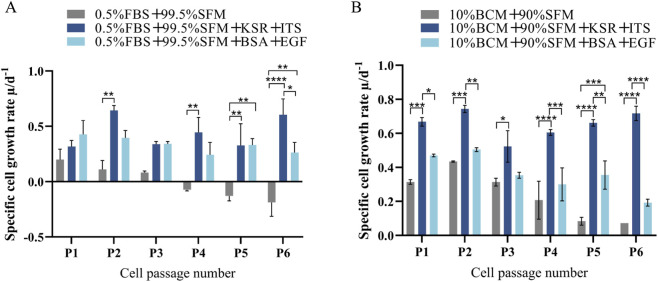
Specific cell growth rate under serum concentration reduction and dual-medium gradient adaptation with different combination treatments. **(A)** Serum concentration reduction adaptation with different substance combinations; **(B)** Dual-medium gradient adaptation with different substance combinations. BCM: Dulbecco’s Modified Eagle’s Medium +10% FBS + Puromycin (Puro). SFM: serum-free medium. KSR: Knockout™ serum replacement. ITS: insulin–transferrin–selenium. BSA: bovine serum albumin. EGF: epidermal growth factor. *P < 0.05, **P < 0.01, and ***P < 0.001.

All the groups were continuously passaged six times, and the specific growth rate of cells was dynamically monitored (Figure 5). The specific growth rate of cells in the adaptation mode control groups gradually decreased with the increase in the passage frequency. The serum concentration reduction in the control group showed growth arrest at the fourth passage. Under Step 5 conditions, the specific growth rate of the experimental group with 10% KSR +1% ITS was significantly higher than that of the control group (*P* < 0.05) and the experimental group with BSA + EGF (*P* < 0.05).

### 3.3 Multi-dimensional evaluation of cell quality

#### 3.3.1 Cell morphology and skeleton integrity

**FIGURE 6:**
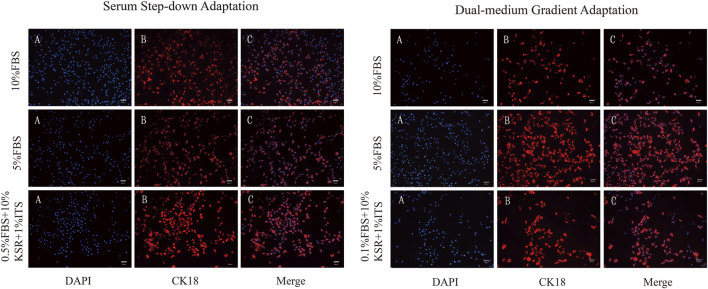
Immunofluorescence images of MAC-T cells. **(A)** Nucleus staining of MAC-T cells; **(B)** Cytokeratin 18 (CK18) staining; **(C)** Combined nucleus and CK18 staining. KSR: Knockout™ serum replacement. ITS: insulin–transferrin–selenium. Scale bar = 200 μm.

To confirm the epithelial characteristics of MAC-T cells overexpressing bovine ferritin under different low-serum conditions, immunofluorescence staining for the expression of CK18 was conducted. When the cell fusion degree reached approximately 70%, fixation and immunofluorescence staining (for CK18) were performed. In all the test conditions for both conditioning modes, that is, serum concentration decreasing and dual-medium gradient adaptations, including basic culture conditions and treatment conditions with KSR + ITS, the cells showed strong positive CK18 fluorescence signals. This confirmed that they maintained the typical epithelial cell skeleton structure (Figure 6).

#### 3.3.2 Stability of cell gene expression during different low-serum stages

**FIGURE 7:**
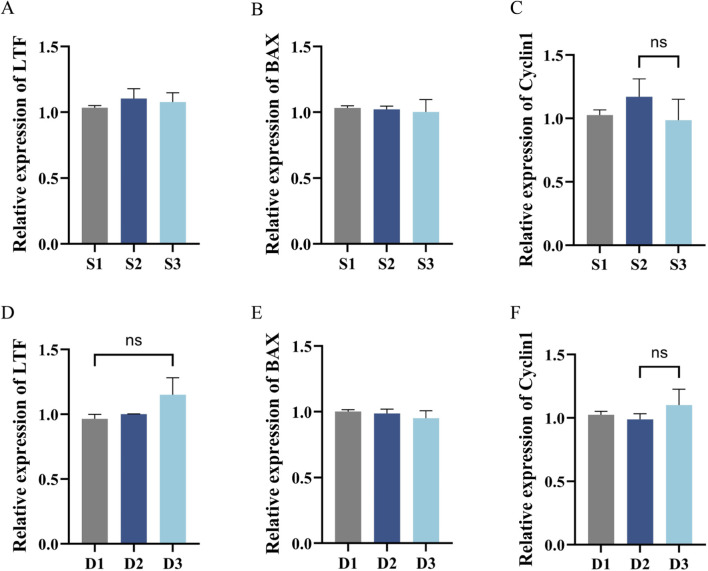
Quantitative reverse transcription polymerase chain reaction (RT-qPCR) for the detection of *LTF*, *BAX*, and *CCND1* expression in MAC-T cells. Serum concentration decreasing acclimation: **(A–C)** Sequentially showed RT-qPCR detection of *LTF*, *BAX*, and *CCND1* gene expression in cells; Two-medium gradient acclimation: **(D–F)** Sequentially showed RT-qPCR detection of *LTF*, *BAX*, and *CCND1* gene expression in cells; S1: 10% fetal bovine serum (FBS) + 90% serum-free medium (SFM) medium; S2: 5% FBS +95% SFM medium; S3: 0.5% FBS +99.5% SFM +10% KSR +1% insulin–transferrin–selenium (ITS) medium; D1: 100% basal control medium (BCM) medium; D2: 50% BCM +50% SFM medium; D3: 10% BCM +90% SFM +10% KSR +1% ITS medium. BCM: Dulbecco’s Modified Eagle’s Medium +10% FBS + Puromycin (Puro). SFM: serum-free medium. KSR: Knockout™ serum replacement. ITS: insulin–transferrin–selenium. *P < 0.05, **P < 0.01, and ***P < 0.001.

The mRNA expression levels of the lactoferrin (*LTF*), apoptosis (BCL2 associated X, *BAX*), and cell cycle (Cyclin D1,*CCND1*) genes in cells at each stage of serum concentration reduction and double-liquid medium gradient low-serum were determined using RT-qPCR. At different low-serum stages, the expression levels of *LTF*, *BAX*, and *CCND1* in the cells showed no significant changes (Figure 7). This indicates that under different serum culture conditions, the expression of core functional genes of the cells, including the genes involved in differentiation/secretion, apoptosis, and proliferation regulation, remained stable, and the physiological state of the cells was retained.

#### 3.3.3 Consistency and ELISA detection of LTF protein expression in different low-serum adaptation steps

**FIGURE 8:**
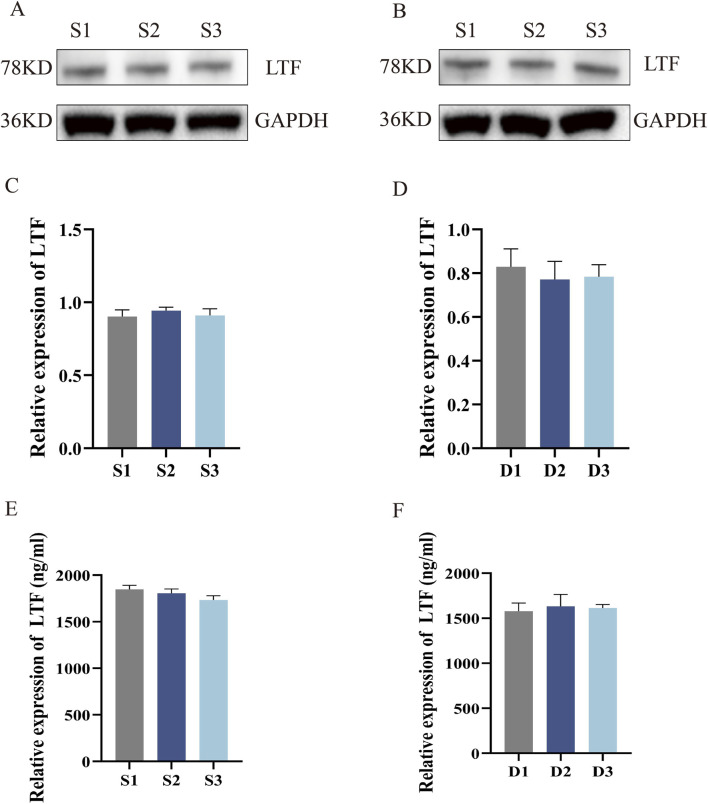
Western blotting and ELISA detection of LTF protein expression in MAC-T Cells (quality evaluation) and their culture medium. **(A)** LTF protein expression in cells adapted via serum concentration reduction; **(B)** LTF protein expression in cells adapted via dual-medium gradient; **(C)** Gray-scale analysis of LTF protein in cells adapted via serum concentration reduction, *n* = 3; **(D)** Gray-scale analysis of LTF protein in cells adapted via dual-medium gradient adaptation, *n* = 3; **(E)** LTF protein expression in the culture medium of cells adapted via serum concentration reduction; **(F)** LTF protein expression in culture medium of cells adapted via dual-medium gradient adaptation; S1: 10% FBS +90% SFM medium; S2: 5% FBS +95% SFM medium; S3: 0.5% FBS +99.5% SFM +10% KSR +1% ITS medium; D1: 100% BCM medium; D2: 50% BCM +50% SFM medium; D3: 10% BCM +90% SFM +10%KSR +1% ITS medium. BCM: Dulbecco’s Modified Eagle’s Medium +10% FBS + Puromycin (Puro). SFM: serum-free medium. KSR: Knockout™ serum replacement. ITS: insulin–transferrin–selenium. *P < 0.05, **P < 0.01, and ***P < 0.001.

The expression of LTF protein in cells at different low-serum stages was assessed via Western blotting using serum concentration reduction and different gradients of dual culture media (Figures 8A,B). The gray-scale analysis of the protein bands indicated no significant differences in the expression of LTF in cells at different low-serum stages and under varying low-serum culture conditions. The expression performance of milk protein in the cells was normal (Figure 8C). Cell supernatants were subjected to ELISA to determine the serum concentration changes, differences in double-culture medium gradients at different low-serum stages, and expression of LTF protein in cells under different culture conditions. Consistent with the Western blotting results, LTF expression levels showed no significant differences among the culture conditions and exceeded 1,500 ng/mL, indicating high lactation performance from the cells (Figures 8E,F).

## 4 Discussion

Considering the serum-free method used to culture HEK293 cells by Jang et al. (2022), two culture modes were designed to explore the adaptation mechanisms of cells to the low-serum environment, including serum concentration reduction culture and dual-medium gradient culture. During both low-serum modes, the cell growth state showed stage specificity. When the serum concentration decreased from 0.5% to 0.1%, some cells floated and became round and shiny. This is related to the reduction of attachment factors in the serum, leading to lose cell adhesion (Lou et al., 2025). This is a typical adaptive response to the low-serum environment. The critical concentration of both modes occurred at Step 5. However, the serum thresholds differed. The critical levels for serum concentration reduction and double-medium gradient low-serums were 1% and 0.5% FBS, respectively. The SFM used in the serum concentration reduction mode had richer nutritional components and better supported cell growth under low-serum concentrations, consistent with the sensitivity of epithelial-like cells to the nutritional environment. In the first four low-serum steps, the cells maintained the typical polygonal morphology of epithelial cells and were stable. In contrast, during the critical concentration stage, the cell morphology became round, the adhesion ability decreased, and the specific growth rate declined (Jang et al., 2022). This reflected the metabolic and phenotypic adjustments in cells to enable adaptation to the low-serum environment.

Low-serum acclimation in cells enables the gradual establishment of an adaptation mechanism to the low-nutrient environment. The cell function and phenotype stability depend on the metabolic adjustment during the continuous passage process. During the serum concentration reduction process, at least three consecutive generations are required to stabilize the cell state (Xie, 2024). Therefore, we designed a slow acclimation procedure in this study. Each acclimation step required three consecutive passages and stable growth before initiating the next stage. In actual acclimation, in the first six steps of the two modes, that is, serum step-down and double-medium gradient adaptations, the cell viability remained >90% and was normal. This confirmed the effectiveness of this procedure. However, at Step 6 (1% BCM +0.1% FBS), although the cells grew, their state was unstable. This further indicates the necessity of continuous stable passages for cells to adapt to the low-serum environment. Cells maintain functional stability at low-serum concentrations only through adequate metabolic adjustment.

During the slow low-serum process, a growth arrest occurred at Step 5 in both modes. This stage became the key bottleneck for cells to adapt to the low-serum environment, suggesting the need to introduce serum substitutes to compensate for the lost serum functions. Substances, such as proteins, insulin, and growth factors, likely partially replace the nutritional and signaling functions of serum (Golshan et al., 2025), providing a theoretical basis for component screening in this experiment. As a clearly defined serum-free preparation, KSR has abundant amino acids, vitamins, and trace elements. It enhances the response of cells to limited growth factors, promotes proliferation, and maintains survival by stabilizing signaling pathways, such as the mitogen-activated protein kinase cascade (Haraguchi et al., 2025). It is a promising substitute for FBS in the cultivation of pig spermatogonial stem cells and human dermal fibroblasts (Gomes et al., 2020; Fayaz and Honaramooz, 2022; Hidayati et al., 2023). BSA maintains osmotic pressure, provides fatty acids, and protects cells against protease damage (Skrivergaard et al., 2023). EGF regulates cell division, proliferation, and metabolism by activating cell surface receptor signaling pathways (Ahmed et al., 2023), while ITS supplements key regulatory factors like insulin and transferrin to regulate metabolism (Zhang et al., 2006). In this study, we screened two combinations, 10% KSR +1% ITS and 1 mg/mL BSA +20 ng/mL EGF. These two combinations represent two distinct strategies for low-serum culture, that is, serum nutrient simulation and basic nutrition plus proliferation regulation. This can clarify the differences in the effects of different strategies on the growth of MAC-T cells. Under Condition 5, the cell growth rate of the 10% KSR +1% ITS group was significantly higher than that of the control and BSA + EGF groups, which indicated that the combination of 10% KSR and 1% ITS was better to meet the growth requirements of cells in a low-serum environment. However, when the serum concentration dropped to the serum-free level, the cells died. This suggests that the current culture system cannot fully replace the serum functionally. This is potentially related to the absence of certain key signaling molecules or adhesion factors in the serum-free environment. Further optimization of the medium components, such as supplementing specific adhesion or growth factors, is required to overcome this limitation (Zhang et al., 2024; Vaz et al., 2024; Pfeifer et al., 2025). In this study, the 10% KSR +1% ITS combination showed a significantly low-serum adaptation effect on the target cells. However, it may not apply to other cell lines. Therefore, developing personalized supplement formulations for specific cell lines is crucial to achieving serum-free culture.

The comparison of the two low-serum modes further highlights the differences in cell adaptation. Mechanistically, the serum concentration reduction mode relies on SFM for direct serum reduction, focusing on cells tolerance to serum deprivation; the double-medium gradient mode uses BCM-SFM mixture to achieve transitional adaptation and reduce environmental stress (Jang et al., 2022). In terms of growth performance, both modes maintained over 90% cell viability and stable growth in the first 4 steps (serum ≥1% FBS or equivalent BCM). However, at Step 5 (0.5% FBS for the former, 10% BCM equivalent to 1% FBS for the latter), the serum concentration reduction mode showed significantly lower growth rate (P < 0.05) and more obvious viability decline, due to lower serum concentration and lack of BCM nutritional compensation. For application potential, cells in the serum concentration reduction mode achieved stable growth as early as Step 4 (1% FBS), and could adapt to 0.5% FBS after adding 10% KSR +1% ITS, with simple operation and controllable cost fitting industrial needs. The double-medium gradient mode with gentler process is suitable for initial low-serum stage but requires additional BCM preparation, leading to higher complexity and cost.

In this study, we did not adopt CHO cells or other cell lines that are widely used in the production of recombinant therapeutic proteins. As a protein naturally expressed and secreted by mammalian mammary epithelial cells, the correct folding, modification, and secretion of LTF depend on the transcriptional regulatory networks and secretory pathways of mammary epithelial cells. As an immortalized bovine mammary epithelial cell line, MAC-T cells retain typical epithelial cell characteristics, verified as CK18-positive, and have the cell-specific mechanisms required for LTF expression (Kwon et al., 2024). This can ensure the efficient and stable synthesis of LTF. The mammary cell-specific promoter regulation, Golgi apparatus processing, and extracellular secretion signaling pathways can guarantee the biological activity and natural properties of LTF. In contrast, CHO cells are derived from hamster ovaries and belong to ovarian epithelial-like cells which lacked the LTF expression regulatory system of mammary epithelial cells (Majumdar et al., 2025; Zhang et al., 2024). In this study, we aimed to establish a low-serum culture system for MAC-T cells stably overexpressing LTF for the large-scale culture of MAC-T cells and *in vitro* production of LTF, such as mammary gland bioreactors (Li et al., 2024). The LTF expressed by MAC-T cells is consistent with natural bovine milk LTF in terms of amino acid sequence and modification patterns, making it more in line with the application needs of LTF in the food and health product fields (Skoog et al., 2024). However, heterologous proteins expressed by CHO cells may have safety concerns, such as immunogenicity when used in the food field, and their culture processes have low compatibility with food-grade protein production (Zeh et al., 2024). MAC-T cells were the optimal choice for this study.

Compared with the low-serum culture processes of commonly used engineering cell lines such as HEK293 cells, Vero cells, and lamb muscle cells, the present study achieved an approximate 60% reduction in total costs with a target concentration of ≤1% FBS, markedly outperforming the cost optimization ranges of 30%–50% for HEK293 cell low-serum culture (Jang et al., 2022), approximately 40% for Vero cell serum-free culture (Posung et al., 2021), and around 25% for lamb muscle cell serum-free culture (Amirvaresi and Ovissipour, 2025). Most low-serum cultures of other cell lines take cell survival and basic proliferation as the core goal, and their generalized serum substitute combinations often require additional redundant components: such as the adhesion factor polylysine needed for HEK293 cells, high-cost growth factors (bFGF for HEK293 cells and recombinant EGF for Vero cells), and myogenic differentiation regulatory factors required for lamb muscle cells (Jang et al., 2022; Posung et al., 2021; Amirvaresi and Ovissipour, 2025). These components not only increase the proportion of substitute costs but also extend the acclimation cycle and raise reagent loss. In contrast, the present study targeted and screened the exclusive substitute combination of 10% KSR +1% ITS (Gomes et al., 2020; Skrivergaard et al., 2023). It not only utilizes KSR to mimic core serum nutritional properties but also uses ITS to precisely supplement the energy supply required for LTF synthesis, eliminating the need for additional unnecessary factors. Meanwhile, the serum dosage was drastically reduced from the traditional 10% to ≤1%, maximizing the reduction of FBS expenditure, which accounts for the largest proportion of the total medium cost, while ensuring the stability of LTF expression and cell viability. This study provides a more industrially applicable technical template for the large-scale culture of engineering cell lines producing functional proteins.

In this study, we found that the serum concentration reduction pattern combined with the 10% KSR +1% ITS supplement was suitable for the stable growth of MAC-T cells under 0.5% FBS conditions, providing a feasible solution for low-serum cultures. However, the serum-free acclimation requires further optimization. In the future, by analyzing the metabolic defects of cells in the serum-free environment, key factors such as essential amino acids could be supplemented to promote the complete elimination of serum from the culture system.

## 5 Conclusion

This study employed two acclimation methods, including serum concentration reduction and dual-medium gradient adaptation to gradually reduce the serum dependence of MAC-T cells with stable expression of LTF. After systematically evaluated the growth characteristics (specific growth rate, cell doubling time, and cell viability) of the cells and the LTF expression stability during the acclimation process, we found that both strategies successfully achieved low-serum adaptation under a 1% FBS condition. At the stage where the serum concentration decreased to 0.5% FBS or the double-medium gradient acclimation reached 10% BCM (equivalent to 1% FBS), adding 10% KSR +1% ITS could effectively promote the cell to increase the specific growth rate (approximately 30%) and effectively promote the cell to grow stably at low-serum concentrations (0.5% FBS/10% BCM). Under the low-serum conditions in each acclimation stage, the cells maintained typical epithelial cell morphology (CK18-positive), and the mRNA transcription levels, protein expression, and secretion levels of LTF remained stable. Therefore, this study established an effective acclimation system for MAC-T cells in a low-serum environment and laid a certain foundation for the low-serum or serum-free large-scale cultivation of MAC-T cells and the *in vitro* production of high-quality LTF.

## Data Availability

The original contributions presented in the study are included in the article/supplementary material, further inquiries can be directed to the corresponding authors.
